# Metabolic effects of the dietary monosaccharides fructose, fructose–glucose, or glucose in mice fed a starch‐containing moderate high–fat diet

**DOI:** 10.14814/phy2.14350

**Published:** 2020-02-05

**Authors:** Lianne M. S. Bouwman, Arie G. Nieuwenhuizen, Hans J. M. Swarts, Rosaria Piga, Evert M. van Schothorst, Jaap Keijer

**Affiliations:** ^1^ Human and Animal Physiology Wageningen University Wageningen The Netherlands

**Keywords:** energy intake, indirect calorimetry, isocaloric diets, liver metabolism

## Abstract

Fructose consumption has been linked to obesity and increased hepatic de novo lipogenesis (DNL). Excessive caloric intake often confounds the results of fructose studies, and experimental diets are generally low‐fat diets, not representative for westernized diets. Here, we compared the effects of dietary fructose with those of dietary glucose, in adult male and female mice on a starch‐containing moderate high–fat (HF) diet. After 5 weeks fattening on a HF high‐glucose (HF‐G) diet, mice were stratified per sex and assigned to one of the three intervention diets for 6 weeks: HF high fructose (HF‐F), HF with equimolar glucose and fructose (HF‐GF), or HF‐G. Bodyweight (BW) and food intake were measured weekly. Indirect calorimetry was performed on week 5; animals were sacrificed in food‐deprived state on week 6. Data were analyzed within sex. BW gain was similar among animals on the HF‐G, HF‐GF, and HF‐F diets. Cumulative food intake was slightly lower in HF‐F animals (both sexes). However, energy expenditure was not affected, or were circulating insulin and glucose concentrations, and hepatic triglyceride levels at endpoint. Hepatic gene expression analysis showed only minor alterations in hexokinase and glycolysis‐related expression in males, and no alterations in sugar transporters, or DNL‐related enzymes. In females, no consistent alterations in hepatic or small intestine gene expression were seen. Concluding, partial or complete replacement of dietary glucose with fructose does not increase caloric intake, and does not affect BW, hepatic triglyceride levels, or insulin concentrations in male and female mice on a moderate high–fat diet.

## Graphical abstract

Effects of dietary fructose are compared with those of dietary glucose and of dietary glucose and fructose in equimolar amounts. The study was performed in adult male and female mice on a starch‐containing moderate high–fat (HF) diet. The partial or complete replacement of dietary glucose with fructose did not increase caloric intake, and did not affect BW, hepatic triglyceride levels, or insulin concentrations in male and female mice on a moderate high–fat diet.

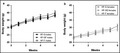

## 1 INTRODUCTION

The worldwide increase in prevalence of obesity is a major health concern (WHO, 2018). The cause of this increase is considered to be multifactorial, with dietary habits and a sedentary life style as two main contributors. In addition, it has been hypothesized in the early 2000s that the increase in obesity and its related health problems are caused by a rise in fructose consumption, in particular by fructose coming from beverages (Bray, Nielsen, & Popkin, 2004). A decade later, Bray *et al.* (still) state that the consumption of sugar‐sweetened beverages is contributing to the high incidence of obesity, and that fructose “has critical adverse effects” (Bray & Popkin, 2014). Others also suggested that fructose exerts detrimental health effects, much worse than caloric equivalents, and state that its effects resemble those of alcohol (Lustig, 2013). However, this theory is controversial, as several research groups suggest that it is not the increased sugar or fructose consumption per se that is contributing to the pandemic of obesity, but the overall high caloric intake (Sievenpiper et al., 2014; Tappy, 2012; White, 2013).

Glucose and fructose are both monosaccharides, have the same molecular weight, yet the structural difference causes the monosaccharides to be absorbed and metabolized differently, for instance, using different specific intestinal transporters. Cellular metabolism differs in the first steps, with glucose breakdown via glycolysis being regulated by phosphofructokinase (PFK), while this step is bypassed in the breakdown of fructose (Michal & Schomborg, 2012; Tappy & Le, 2010).

Activity of PFK depends on the energy level of the cell. When the latter is high, ATP and citrate levels are high, inhibiting PFK activity (Tappy & Le, 2010). KHK activity, however, is not regulated by the energy status of the cell (Tappy & Le, 2010). Therefore, it is believed that in fed conditions fructose is directed toward de novo lipogenesis (DNL), whereas glucose is directed to glycogenesis (Lustig, 2010). This is used as explanation for hepatic lipid accumulation in studies with fructose supplementation in the drinking water. Others, however, showed that fructose also contributes to glycogenesis (Delgado et al., 2013). Scientific evidence underlying the potential adverse health effects of fructose is often derived from animal studies, yet the results of these studies are often difficult to translate to the human situation. Fructose in animal studies is generally given in very high doses (up to 60 energy percent (en%) from fructose) and the animals are on a low‐fat dietary background. In contrast, the contribution of fructose to total energy in the human diet is about 9% (median intake in The Netherlands (Sluik, Engelen, & Feskens, 2015) and the United States (Sun, Anderson, Flickinger, Williamson‐Hughes, & Empie, 2011)), and 17.8% for the 95th percentile fructose consumption (Sun et al., 2011). Moreover, the low‐fat dietary background in most animal studies is also not representative for the human situation; for example, in The Netherlands the median contribution of fat to total energy intake in adults is approximately 34% (van Rossum, Fransen, Verkaik‐Kloosterman, Buurma‐Rethans, & Ocké, 2011). In addition, most animal studies investigating fructose use young, normal weight animals. However, this is scarcely representative of the human population that is increasingly overweight and obese. Therefore, a period of fattening before the start of an animal study will result in a better representation of the current human situation.

There is accumulating evidence that sex differences exist in the response to fructose. Several human intervention studies have shown that the effects of fructose are attenuated in females. Acute fructose consumption leads to higher uric acid and lactate responses in males than in females (Panda et al., 1991) and enhanced hepatic DNL stimulation (Tran et al., 2010). In addition, the effects of fructose seem blunted in females compared with males, as seen with 6 days of fructose overfeeding (Couchepin et al., 2008) and after a 6‐week fructose diet intervention with fructose supplementation (Bantle, Raatz, Thomas, & Georgopoulos, 2000). Several studies in animal models focus mainly on the effects of long‐term fructose intake on liver health. Female mice are more susceptible to liver damage than males when their drinking water is supplemented with 30% fructose for 16 weeks, even though males have higher weight gain (Spruss et al., 2012). Liver weight and visceral adiposity increase more in female rats compared with their male counterparts with a fructose intervention of 9 weeks; however, in males, insulin sensitivity and blood pressure are affected (Koricanac et al., 2013). Underlying alterations in hepatic metabolism by 2 weeks fructose supplementation are more severe in females, and mediated by hepatocyte nuclear factor 4 (HNF4), whereas in males the reduction in peroxisome proliferator‐activated receptor α (PPARα) causes the alterations in liver (Vilà et al., 2011). However, in all these studies fructose was provided in the drinking water, causing an increased liquid intake and a decreased food intake, which cumulatively resulted in an alteration in the ratio of carbohydrate intake to fat intake, as well as in a net increase in caloric intake in the fructose groups. Therefore, the effects cannot simply be regarded as the effects of fructose as such, as they may result from a higher caloric intake.

The aim of this study was, therefore, to elucidate the effects of dietary fructose, glucose, and glucose–fructose in a 1:1 ratio (all in dietary isocaloric amounts) on bodyweight development and hepatic gene expression, in mice on a moderate high–fat diet. Fructose and glucose were administered as part of the pelletized diet, to circumvent the confounding effects of overconsumption of fructose in the drinking water. The diet had a moderate high–fat content and contained starch, to provide a relevant dietary context to the monosaccharides. The diet with glucose and fructose in a 1:1 ratio, representing the common dietary sugar sucrose, was used because of dietary relevance, and because an interaction of glucose and fructose on gene expression in the liver has been reported (Hamblin, Ozawa, Jefferds, & Mariash, 1989). Moreover, the diets were administered to both males and females to study whether the effects of this intervention is affected by sex, and in animals exposed to a moderate high–fat diet before the intervention, for the induction of a metabolic phenotype that is more representative of an individual, currently in a Western society.

## 2 MATERIALS AND METHODS

The experiment was performed according to the Dutch Animal Experimentation Act (1996). The experimental protocol was approved by the Animal Welfare Committee of Wageningen University, Wageningen, The Netherlands (DEC 2010115.d).

### 2.1 Animals & experimental procedure

Thirty male and thirty female C57BL/6JOlaHsd mice of 9 weeks of age were purchased from Harlan Laboratories (Horst, The Netherlands). Mice were individually housed in Makrolon II cages with standard bedding of wood chips and shavings, with ad libitum access to food and water and were maintained under controlled environmental conditions (temperature 21 ± 1°C; relative humidity 52.5 ± 2.5%; 12 hr–12 hr light–dark cycle, lights on at 5.00 hr). Animals were fed a semisynthetic moderate high–fat (35.9 en% fat) high‐glucose diet (HF‐G; Table 1) obtained from Research Diet Services B.V. (Wijk bij Duurstede, The Netherlands). This diet is an adaptation of the published BIOCLAIMS high‐fat diet (Hoevenaars et al., 2012), which is known for the induction of insulin resistance (Voigt, Agnew, Schothorst, Keijer, & Klaus, 2013). After 5 weeks of fattening, the animals were stratified by bodyweight and assigned to one of three dietary groups (*n* = 10 per group, per sex): a moderate high–fat high‐glucose (HF‐G) diet, a moderate high–fat glucose and fructose (HF‐GF) diet, or a moderate high–fat high‐fructose (HF‐F) diet; and these diets only differed in their monosaccharides composition. The detailed dietary compositions can be found in Table 1. Given the monosaccharide content, the diets contained: 28.6 en% glucose and 0 en% fructose (HF‐G), 14.3 en% glucose and 14.3 en% fructose (HF‐GF), and 0 en% glucose and 28.6 en% fructose (HF‐F). All diets contained starch (15.3 en%). The calculation of the required group size was based on past experience with the detection of statistically significant diet‐induced alterations in molecular markers. The dietary intervention lasted for a total of 6 weeks, and bodyweight and food intake were determined weekly in the morning.

**Table 1:** Composition of the intervention diets. Ingredients are given in gram/ kilogram of diet. All diets are moderate high–fat and isocaloric

	Moderate‐high fat–glucose (HF‐G)	Moderate‐high fat–glucose–fructose (HF‐GF)	Moderate‐high fat–fructose (HF‐F)
Casein	225	225	225
Wheat starch	172	172	172
Dextrose	322.5	161.25	0
Fructose	0	161.25	322.5
Lipids^a^	180	180	180
Cholesterol	0.097	0.097	0.097
Cellulose	50	50	50
Mineral mixture	35	35	35
Vitamin mixture	10	10	10
Choline bitartrate	2.5	2.5	2.5
L‐Cysteine	3	3	3
Total	1,000	1,000	1,000
Total energy (kcal)	4,510	4,510	4,510
Protein en%	20.2	20.2	20.2
CHO en%	43.9	43.9	43.9
Fat en%	35.9	35.9	35.9

a The lipid fraction contained 18% cocos oil, 70% sunflower oil, and 12% flaxseed oil (wt/wt) to ensure a health fatty acid profile, as in the BIOCLAIMS diet [27].

### 2.2 Indirect calorimetry

On week 5 of the intervention, animals were placed into a PhenoMaster Indirect Calorimetry System (TSE Systems, Bad Homburg, Germany). Rates of oxygen consumption and carbon dioxide production were measured for each animal once every 12 min. O_2_ consumption, CO_2_ production, and activity were measured as previously described (Hoevenaars et al., 2013). Energy expenditure was calculated every 12 min (previously described (Hoevenaars et al., 2013)) and expressed as kcal/h. The average of all the energy expenditure values was taken and multiplied by 24 to obtain the energy expenditure in kcal/day.

### 2.3 Sacrifice

At the end of the 6th week of the intervention, animals were food deprived for at least 2 hr during the light phase. A tail incision was made and blood glucose concentrations were measured with a Freestyle blood glucose system (Abbott Diabetes Care, Hoofddorp, The Netherlands); no anesthesia was used to prevent effects on blood glucose concentrations (Constantinides, Mean, & Janssen, 2011). Blood was collected in Microvette CB 300 EDTA tubes (Sarstedt, Nürnberg, Germany) to obtain plasma samples by centrifugation at 2000*g*. Immediately afterwards, the animals were anesthetized with a mixture of 5% isoflurane in 1:1 gas mixture of nitrous oxide and oxygen for 1 min, and decapitated. Liver and gonadal fat pads (epididymal fat pad in males and fat pads around uterus in females) were collected, weighed, snap frozen in liquid nitrogen, and stored at −80°C. The small intestine was collected, cut open longitudinally, rinsed, and scraped. Cell scrapings were snap frozen in liquid nitrogen, and stored at −80°C.

### 2.4 Plasma measurements

Insulin concentrations were analyzed in plasma using a Mouse insulin (TMB) ELISA kit (Biovendor GmbH, Kassel, Germany) according to the manufacturer's instructions. Plasma triglyceride (TG) concentrations were measured using an Instruchemie Triglyceride Liquicolor kit (HUMAN, Wiesbaden, Germany). HOMA‐IR was calculated from levels of insulin and blood glucose, using a C57BL/6J mice‐adjusted factor of 14.1, as previously described (Dijk et al., 2013).

### 2.5 Liver measurements

Liver tissue was defrosted and dissolved in Tris‐EDTA buffer (20 mg/ml). The TG level was determined using an Instruchemie Triglycerides Liquicolor kit (HUMAN) according to the manufacturer's instructions. Input was corrected for wet tissue weight. Liver glycogen levels were determined according to a method previously described (Krisman, 1962). Briefly, liver tissue (caudate lobe) was grinded and dissolved in chloric acid solution (7%). Samples were centrifuged at 830*g* for 15 min (4°C) and the supernatant was stored at −80°C for 1.5 hr. Petroleum ether was added to the frozen sample and after shaking, the aqueous layer was collected and stored on ice. A total amount of 260 *μ*l reagent solution (0.90 M calcium chloride; 1.31 mM iodine; and 11.4 mM potassium iodine) was added to 10 *μ*l sample. After 10 min incubation, the absorbance was measured at 460 nm.

### 2.6 Gene expression analysis

Liver or intestinal tissue was grinded and homogenized in liquid nitrogen. Subsequently, RNA was isolated using a RNeasy Mini Kit (Qiagen, Venlo, The Netherlands) according to the manufacturers’ instructions. RNA yield and purity were evaluated using a Nanodrop Spectrophotometer (Isogen Life Science, Maarssen, The Netherlands) and RNA integrity was measured on the Experion automated electrophoresis system (Bio‐Rad, Veenendaal, The Netherlands). cDNA was synthesized using the iScript cDNA synthesis kit (Bio‐Rad). Reverse‐transcription quantitative real‐time polymerase chain reaction (RT‐qPCR) experiments were performed using iQ SYBR Green Supermix (Bio‐Rad). As regard the standard curves, serial dilutions of pooled cDNA from all samples were made. Samples were tested in duplicate; male and female samples were tested in separate runs. An overview of the target genes, primer sequences, and annealing temperatures can be found in Table 2. The expression of target genes was normalized against the geometrical mean of the reference gene(s), and normalized against the mean of the glucose group set at 1.0. For intestinal gene expression, Ribosomal protein S15 (*Rps15*) and Calnexin (*Canx*) were used as reference genes. As regard the hepatic gene expression, *Rps15* and Hypoxanthine phosphoribosyltransferase 1 (*Hprt1*) were used as reference genes for the expression of *Slc2a2, Slc2a5*, *Hk1*, *Hk2*, *Hk4*, *Khk*, *Aldob*, *Tkfc*, *Gapdh,* and *Tpi1*; *Canx* was used as reference gene for DNL genes acetyl‐CoA carboxylase beta (*Acacb*), fatty acid synthase (*Fasn*), elongation of long‐chain fatty acids family member 6 (*Elovl6*), and stearoyl‐coenzyme A desaturase 1 (*Scd1*) as well as microsomal triglyceride transfer protein (*Mttp1*), a protein involved in hepatic lipid export.

**Table 2:** Target genes, primer sequences, and annealing temperatures in RT‐qPCR

Gene	Forward Primer 5′−3′	Reverse Primer 5′−3′		Annealing Temperature (°C)
*Acacb*	TCTTCACGTTCAGAGCGAGGGAT	ATCTTGTGGTTGGCACAGGGCA	55.6	
*Aldob*	TACCAGATACAACCCCAATAGTCAGG	TCAACTCATTCTCTACACTCCATAGC	61.5	
*Canx* a	GCAGCGACCTATGATTGACAACC	GCTCCAAACCAATAGCACTGAAAGG	60.0	
*Elovl6*	GGCACTAAGACCGCAAGGCA	GCTACGTGTTCTCTGCGCCT	60.0	
*Fasn*	AGTTAGAGCAGGACAAGCCCAAG	GTGCAGAGCTGTGCTCCTGA	60.0	
*Gapdh*	AAGGCTGTGGGCAAGGTCATC	CGAAGGTGGAAGAGTGGGAGTTG	61.5	
*Hk1*	ACGCTCGGTGCCATCTTGAAC	CCTTGCCACTGCCACTCTCC	61.5	
*Hk2*	ATCAAAGAGAACAAGGGCGAGGAG	GCGGAGGAAGCGGACATCAC	61.5	
*Hk4*	CCTGGGCTTCACCTTCTCCTTC	CCTCACATTGGCGGTCTTCATAG	61.5	
*Hprt1* a	TGACACTGGTAAAACAATGCAAACTTTG	GAGGTCCTTTTCACCAGCAAGCT	61.5	
*Khk*	GCAGCGGATAGAGGAGCACAATG	CCAGGCACAGACAAGCGTAGC	61.5	
*Mttp1*	GTGGAGGAATCCTGATGGTGA	TGATCTTAGGTGTACTTTTGCCC	61	
*Rps15* a	CGGAGATGGTGGGTAGCATGG	ACGGGTTTGTAGGTGATGGAGAAC	60	
*Scd1*	TCATGGTCCTGCTGCACTTGG	CTGTGGCTCCAGAGGCGATG	60	
*Slc2a2*	CACACCAGCATACACAACACCAG	GGACACAGACAGAGACCAGAGC	61.5	
*Slc2a5*	TCCTCCTCCTCCCGTTCTTTCC	CTTCTCAGCCTCATCCTCCTTCC	61.5	
*Slc2a7*	TCGGTGGTGAGGACAGAGATTG	AGCAGCAGTGAGGATACAGACG	61.5	
*Slc5a1*	TCGTCATCTACTTCGTGGTGGTG	CCTGCGGCTGCTCCTGTG	61.5	
*Tkfc*	GGGCAGCAGCACAGGAGTTC	AGGATGGCACGGAAGATGGC	61.5	
*Tpi1*	GGACTGGCAAGACGGCAAC	GCAGGCAGGTAGAGGGATGG	61.5	

a Transcripts used for normalization.

### 2.7 Statistical analysis

Statistical analysis was performed using GraphPad Prism version 5.04 (Graphpad Software, San Diego, USA). Comparisons were made between the different diets within each sex. Data were checked for normality by a D’Agostino & Pearson omnibus normality test. If data were not normally distributed, they were log converted and rechecked for normality. A one‐way ANOVA with post hoc Tukey comparison was used for normally distributed datasets. In case of not‐ normally distributed data or unequal variances, a Kruskal–Wallis test was performed, followed by a Dunn's Multiple comparison test. Bodyweight and RER curves were analyzed by two‐way ANOVA. *p*‐values <.05 were considered statistically significant.

## 3 RESULTS

**Figure 1:**
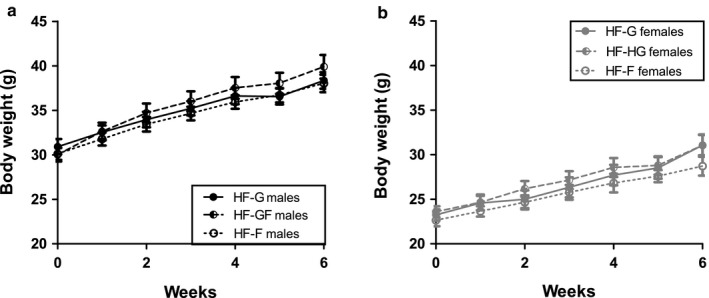
Bodyweight development. Bodyweight development during the intervention period for males (a) and females (b) when giving a 35.9 en% high‐fat diet containing 28.6 en% of glucose (HF‐G, solid dots and lines), 14.3 en%‐14.3 en% glucose and fructose (HF‐GF, half‐open dots and striped lines), or 28.6en% fructose (HF‐F, open dots and dotted lines). Values are expressed as mean ± *SEM*; *n* = 10

Bodyweight was not significantly different among animals on a moderate high–fat diet rich in glucose (HF‐G), fructose (HF‐F), or glucose‐fructose in a 1:1 ratio (HF‐GF), neither for males (Figure 1a) nor for females (Figure 1b). Only in male mice, the interaction time x diet was significant: HF‐GF males gained more bodyweight than HF‐G males or HF‐F males. Cumulative caloric intake was significantly higher in HF‐G animals compared with HF‐F animals in both sexes (Table 3). As diets were isocaloric, differences in caloric intake were due to a difference in food intake. Animals on the HF‐GF diet had an intermediate food intake.

**Table 3:** Food intake, energy expenditure, RER, and organ weights. The cumulative caloric intake is calculated over the 6 weeks experimental period

		Cumulative feed intake (g)	Energy expenditure (kcal/day)	Average RER (dark phase)	Average RER (light phase)	Gonadal fat pad weight (g)	Liver weight (g)
Males	HF‐G	149 ± 10^a^	12.55 ± 0.53	0.86 ± 0.02	0.80 ± 0.03	1.95 ± 0.29	1.43 ± 0.15
	HF‐GF	146 ± 8^a,b^	13.10 ± 0.61	0.86 ± 0.02	0.83 ± 0.03	2.12 ± 0.33	1.52 ± 0.21
	HF‐F	140 ± 8^b^	12.82 ± 0.41	0.87 ± 0.02	0.82 ± 0.03	2.07 ± 0.40	1.50 ± 0.12
Females	HF‐G	156 ± 10^x^	11.93 ± 0.66	0.88 ± 0.06	0.82 ± 0.06	1.35 ± 0.46	1.10 ± 0.15
	HF‐GF	150 ± 13^x,y^	11.93 ± 0.52	0.90 ± 0.04	0.82 ± 0.04	1.33 ± 0.50	1.13 ± 0.12
	HF‐F	138 ± 19^y^	11.69 ± 0.59	0.91 ± 0.04	0.85 ± 0.05	1.13 ± 0.34	1.14 ± 0.16

Note

The mean energy expenditure is calculated over a 24‐hr period in the indirect calorimetry system. The mean respiratory exchange ratio (RER) is calculated over a 10‐hr (light phase) or 12‐hr (dark phase) period. Gonadal fat pad weight and liver weight were measured at sacrifice. Different superscript letters indicate significant differences (*p* < .05), data are analyzed within sex. Values are expressed as mean ± *SD*, *n* = 10.

**Figure 2:**
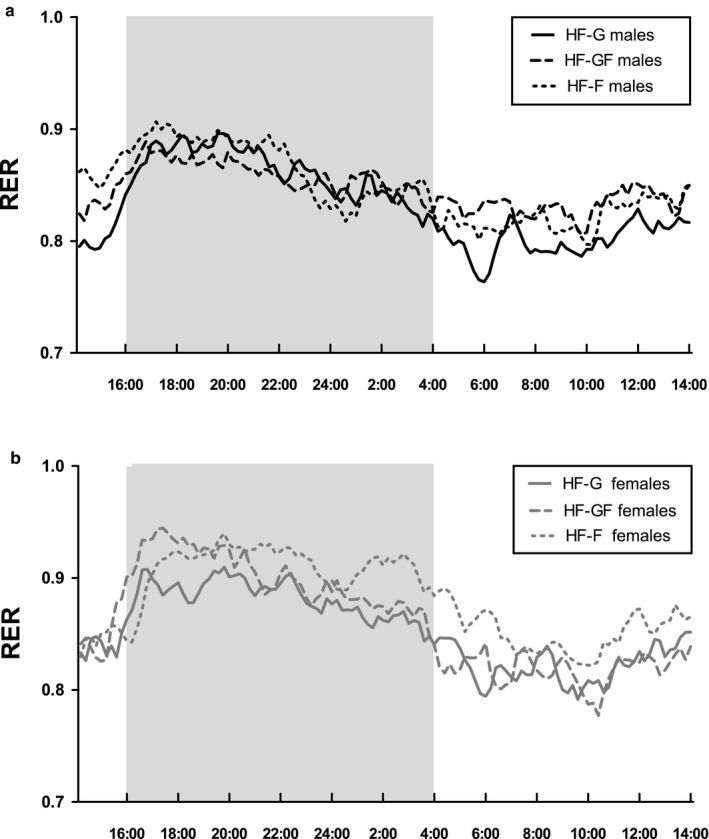
Mean Respiratory Exchange Ratio (RER) over a 24‐hr period. RER was measured by indirect calorimetry, in male (a) and in female (b) mice. The grey shaded area represents the dark phase (when animals are active), and the white shaded areas represent the light phase (when animals are inactive). Solid lines represent HF‐G, striped lines represent HF‐GF, and dotted lines represent HF‐F; lines show mean of the group (*n* = 10)

Indirect calorimetry measurements in week 5 showed that mean RER values were higher in the dark phase than in the light phase in both males (Figure 2a, Table 3) and females (Figure 2b, Table3), indicating relatively more carbohydrate metabolism during the dark phase and more lipid metabolism in the light phase. However, no effect of diet on RER was found in either sex (Figure 2, Table 2). Twenty‐four hour energy expenditure was not affected by the diet, in males as well as females (Table 2).

**Figure 3:**
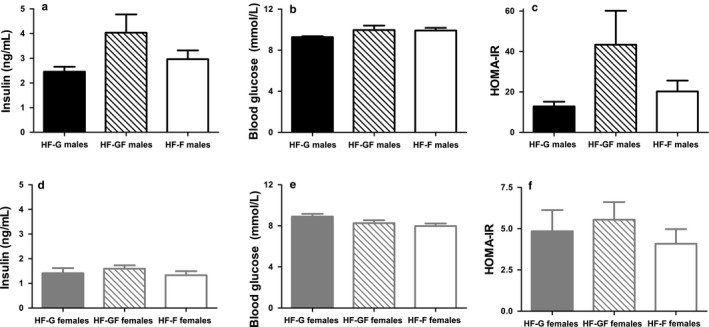
Plasma insulin, blood glucose, and HOMA ‐IR. Plasma insulin concentrations in male (a) and female (d) mice, blood glucose concentrations in males (b) and females (e), and HOMA‐IR in males (c) and females (f). Solid bars represent HF‐G diet, striped bars represent HF‐GF, and white bars represent HF‐F. Values are expressed as mean ± *SEM*, *n* = 10 or *n* = 8 (HF‐G males insulin and HF‐G males HOMA‐IR)

Gonadal fat pad weight was similar among HF‐G, HF‐GF, and HF‐F males, suggesting no difference in adiposity at the end of the intervention (Table 3). Gonadal fat pad weight was also similar among HF‐G, HF‐GF, and HF‐F females. Liver weight was not affected by diet in either sex. In males, plasma insulin concentrations (*p* = .11) and blood glucose concentrations (*p* = .20) did not differ significantly among groups (Figure 3a and b). As a consequence, HOMA‐IR was not significantly different (*p* = .11; Figure 3c). For the females, plasma insulin concentrations were not different among the diets (Figure 3d; *p* = .42), and although a trend was observed for the HF‐F females to have a lower blood glucose concentrations than the HF‐G females (Figure 3e), this effect was not significant (*p* = .054). HOMA‐IR index was not significantly affected by the diet in females (Figure 3f; *p* = .41). Overall, this suggests no effect on glucose homeostasis by the diet in both sexes.

**Figure 4:**
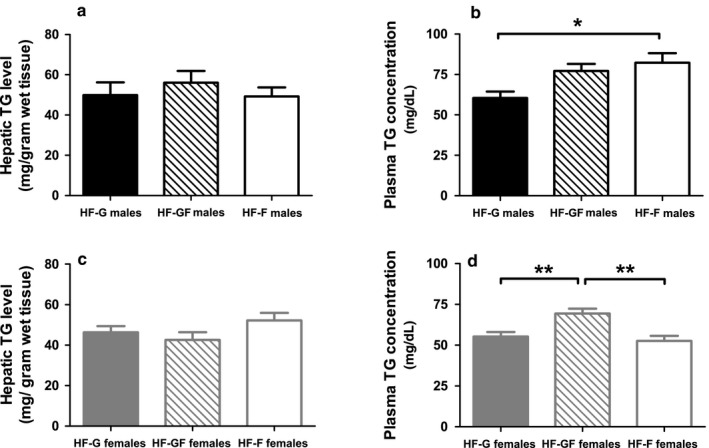
Plasma TG concentrations and hepatic TG levels. Plasma TG concentrations in male (a) and female (c) mice. Liver TG levels in males (b) and females (d). Solid bars represent HF‐G diet, striped bars represent HF‐GF, and white bars represent HF‐F. Values are expressed as mean ± *SEM*, *n* = 8 for the plasma measurement in the HF‐G male group, *n* = 10 for all other groups. *p < .05; **p < .01

Plasma TG concentrations were significantly higher in HF‐F males compared with HF‐G males, with intermediate TG concentrations in HF‐GF male mice (Figure 4a). HF‐GF females had significantly higher plasma TG concentrations than HF‐G and HF‐F females (Figure 4c). In contrast to plasma TG concentrations, total hepatic TG content was not affected by diet (Figure 4b for males, d for females). Hepatic glycogen levels did not differ among the diets in both sexes (data not shown).

**Figure 5:**
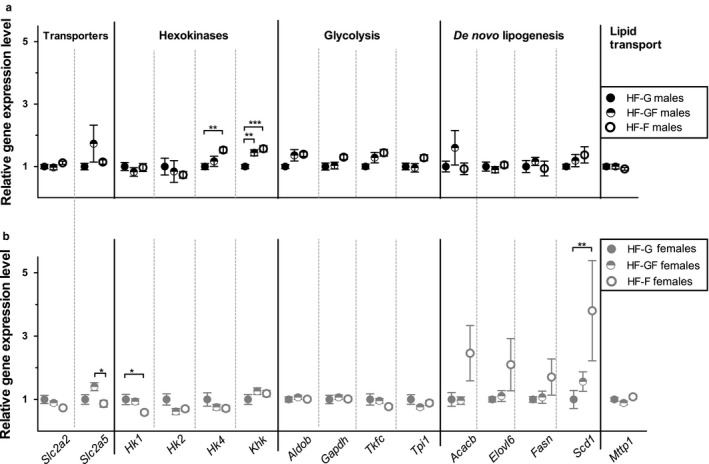
Hepatic gene expression. Expression of enzymes involved in monosaccharide transport, initial glucose conversion (hexokinases), glycolysis, de novo lipogenesis, and lipid transport in liver tissue for (a) male and (b) female mice. *Acacb*: Acetyl‐CoA carboxylase beta; *Aldob*: Aldolase B; *Elovl6*: Elongation of long‐chain fatty acids family member 6; *Fasn*: Fatty acid synthase; *Gapdh*: Glyceraldehyde‐3‐phosphate dehydrogenase; *Hk*: Hexokinase; *Khk*: Ketohexokinase; *Mttp*: Microsomal triglyceride transfer protein; *Scd1*: Stearoyl‐coenzyme A desaturase 1; *Slc2a*: Solute Carrier Family 2; *Tkfc*: *T*riokinase, FMN cyclase; *Tpi*: Triose phosphate isomerase. Solid dots represent HF‐G diet, half‐open dots represent HF‐GF, and open dots represent HF‐F. Values are expressed as mean ± *SEM*, *n* = 9–10. *p < .05; **p < .01; ***p < .001

The mRNA expression of the fructose‐related enzyme ketohexokinase (*Khk*) was increased in both fructose‐fed male groups (HF‐GF and HF‐F) (Figure 5a). Hexokinase 4 (*Hk4*) was the only hexokinase for which the expression was significantly increased in the HF‐F males compared with HF‐G males, while the expression of other hexokinases was not affected. The fructose content of the diet did not alter the hepatic expression of monosaccharide transporters (*Slc2a2* and *Slc2a5*), DNL‐related enzymes (*Acacb, Elovl6, Fasn* and *Scd1*), and *Mttp1* in males; of the glycolysis‐related enzymes (*AldoB, Gapdh, Tkfc*, and *Tpi*) only *AldoB* expression was significantly higher in HF‐F males than in HF‐G males. A significant upregulation of *Slc2a5* was found in the HF‐GF females compared with HF‐F females (Figure 5b). *Hk1* was downregulated in the HF‐F females compared with the HF‐G females and HF‐GF females. The hepatic expression of other kinases (*Hk2*, *Hk4,* and *Khk*) and *Slc2a2* was not affected by the fructose content of the diet in females, or were glycolysis‐related enzymes or *Mttp1*. DNL‐related enzyme expression was not significantly altered, except for a higher *Scd1* expression in HF‐F females compared with HF‐G females.

**Figure 6:**
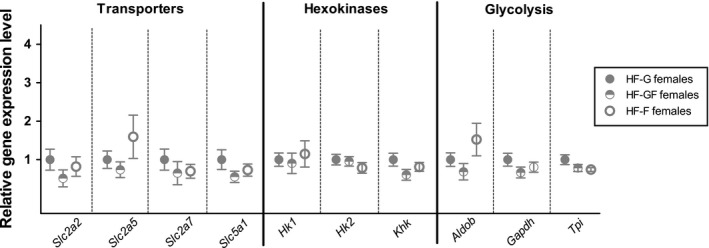
Intestinal gene expression in female mice**.** Expression of enzymes involved in monosaccharide transport, initial glucose conversion (hexokinases), and glycolysis in the intestinal tissue of female mice. *Aldob*: Aldolase B; *Gapdh*: Glyceraldehyde‐3‐phosphate dehydrogenase; *Hk*: Hexokinase; *Khk*: Ketohexokinase; *Slc2a*: Solute Carrier Family 2; *Slc5a*: Solute Carrier Family 5; *Tpi*: Triose phosphate isomerase. Solid dots represent HF‐G diet, half‐open dots represent HF‐GF, and open dots represent HF‐F. Data were with a one‐way ANOVA on original (*Hk2*, *Aldob*, *Gapdh*, and *Tpi*) or log‐transformed data (*Slc2a2*, *Slc2a5*, *Slc2a7*, *Slc5a1*, *Hk*, and *Khk*). Values are expressed as mean ± *SEM*, *n* = 10

Finally, intestinal gene expression was studied in the females. No significant changes were observed in the expression of sugar transporters (*Slc2a2, Slc2a5, Slc2a7,* and *Slc5a1*), hexokinases, or glycolysis‐related enzymes (Figure 6).

## 4 DISCUSSION

Animals fed a Westernized moderate high–fat diet in which half or all of the dietary monosaccharide glucose was replaced with fructose did not show altered HF‐induced bodyweight or adiposity, neither in males nor in females. Nevertheless, plasma TG concentrations were affected by the fructose content, as HF‐F males had higher TG concentrations than HF‐G males, and HF‐GF females had higher plasma TG concentrations than HF‐G and HF‐F females. Liver TG and glycogen levels were not altered by the dietary fructose. From these data we conclude that medium‐ to long‐term isocaloric replacement of dietary glucose with dietary fructose does not affect body composition or liver energy stores. These data are in line with a meta‐analysis in humans, isocaloric studies concluding that fructose has no more effect on bodyweight than other carbohydrates (Sievenpiper et al., 2012). The same group reported that fructose intake, compared with glucose intake, can increase circulating uric acid and TG concentrations, but does not worsen insulin concentrations, markers of fatty liver, or atherogenic aspects of the lipid profile (Sievenpiper et al., 2014). Fructose may even have positive effects on bodyweight and blood pressure in human intervention studies (Sievenpiper et al., 2014). This fully agrees with our observation that plasma TG concentrations were increased by the fructose content in the diet, especially in the combined fructose–glucose exposure. Nevertheless, postprandial plasma TG is particularly influenced under hypocaloric conditions, and not in case of isocaloric replacement, as was shown in another meta‐analysis of human intervention trials (Wang et al., 2014).

Here, we chose to use an intervention period of 6 weeks. For the analysis of hepatic metabolism and respiratory exchange ratio measurements, as performed with indirect calorimetry, this period suffices as differences will arise immediately. In addition, in this mouse strain, an intervention period of 5 weeks was sufficient for stabilization of bodyweight upon switches between high‐ and low‐fat diet (Hoevenaars et al., 2013), and upon moderate caloric restriction of medium‐high–fat diets (Duivenvoorde, Schothorst, Bunschoten, & Keijer, 2011; Palm, Schram, Swarts, Schothorst, & Keijer, 2017). Therefore, we consider the intervention period as sufficiently long, although it should be emphasized that our conclusions on BW and food intake are for a medium‐to long‐term period.

Our data are in striking contrast to other studies that show increased adiposity, increased liver weight, and increased hepatic TG levels resulting from fructose intake. In the large majority of cases, these effects can be explained by excess caloric intake, in particular due to supplementation with fructose via the drinking water or the lack of an adequate control group (Koricanac et al., 2013; Spruss et al., 2012; Vilà et al., 2011). Effects of fructose feeding mimic those of high‐fat feeding (Ren et al., 2012; Song et al., 2012). In addition, fructose can exaggerate the obesogenic effects of high‐fat diet and promote insulin resistance (Crescenzo et al., 2015, 2014). It is not clear whether these effects are fructose specific, or that similar effects would be seen with glucose. Indeed, also maltodextrin exaggerates the effects of a high‐fat diet (Mei et al., 2014). With similar caloric intake, fructose administered via the drinking water gives worse metabolic outcomes than glucose administered via the drinking water in animals on a high‐fat diet (Softic et al., 2017). The overall macronutrient intake was also shifted to some extend in that study (Softic et al., 2017). Nevertheless, overfeeding with fructose beverages in overweight human subjects leads to an increase in visceral adipose tissue and more DNL than overfeeding with glucose beverages (Stanhope et al., 2009). In our study, fructose and glucose were administered as part of the pelletized diet in an isocaloric manner, to circumvent the confounding effects of overconsumption of fructose in the drinking water, and to prevent shifts in macronutrient intake. Although overconsumption and shifts in macronutrient intake were prevented, the fructose diet displayed a significantly lower energy intake in both sexes: HF‐F males consumed 6% less than HF‐G males, whereas HF‐F females consumed 12% less than HF‐G females. Our data contrast with a study where normal‐fat diets with 18% of energy from fructose or from glucose were to young male mice, resulting in increased bodyweight, liver weight, and fat mass by fructose compared with glucose (Rendeiro et al., 2015). Of importance, also the absolute caloric intake was higher in the fructose group, despite isocaloric amounts of the sugars in the experimental diets. The overall lower caloric intake may have had some protective influence in our study. Taken together, outcomes of studies seem to be affected by overall energy intake and by manner of fructose administration (as part of the diet of in a liquid).

To substantiate potential functional differences among fructose, glucose–fructose, and glucose, hepatic gene expression was analyzed, which showed minor changes. The upregulation of KHK in HF‐GF and HF‐F compared with the HF‐G was significant in males, and may be expected as KHK is the first enzyme metabolizing cellular fructose. The limited effect size in other genes may be due to the timing of the analysis: lipogenesis‐related gene expression in liver was upregulated after 5 days of fructose feeding compared with a starch control, but the difference did not persist after 45 days of fructose feeding (Polakof et al., 2016). Alternatively, because the animals were killed in the daytime, 2 hr after removal of the diets, they were possibly in the postprandial state, which may have limited the gene expression effects of fructose. However, this argument is counteracted by the observed upregulation of *Slc5a2* in HF‐GF–fed males and females. The absence of pronounced gene expression effects is in agreement with the absence of differential effects of the monosaccharides on body and liver composition.

Our results did show increased plasma TG concentrations with fructose in the diet (Figure 4). Because hepatic gene expression did not indicate an increase in DNL, these increased concentrations may result from decreased clearance. In fact, decreased lipid clearance can be caused by sucrose (Grant, Marais, & Dhansay, 1994) and fructose (Jeppesen et al., 1995). Increased circulating TG with fructose in the diet was also seen in human males (Bantle et al., 2000; Goff, Whyte, Samuel, & Harding, 2016), but not in females (Bantle et al., 2000). Moreover, as in our study, no differential effects on hepatic TG levels were seen in humans with 25 energy percent from glucose or from fructose, neither with isocaloric feeding nor hypocaloric feeding (Johnston et al., 2013). The absence of a difference between glucose and fructose, but higher hepatic TG levels in the hypercaloric condition compared with the isocaloric condition confirm the notion that it is particularly the energy intake, rather than specific effects of fructose compared with glucose that results in the adverse metabolic effects. Indeed, a recent human study showed also overall lack of effects by high fructose intake (Smajis et al., 2019).

In summary, our physiological data indicate that, within an isocaloric pelletized diet, in the medium‐long–term fructose does not result in worse physiological effects than glucose or glucose–fructose.

## CONFLICT OF INTEREST

None declared.

## AUTHOR CONTRIBUTIONS

JK, AN, and EvS designed the experiment; HS and AN carried out the animal experiment; RP and AN performed the laboratory experiments; LB, EvS, and JK interpreted the results; LB drafted the manuscript; EvS and JK revised the manuscript.
